# Engineering Nanomaterials for Next-Generation Electrochemical Food Safety Sensors: A Comprehensive Review

**DOI:** 10.3390/ma19102170

**Published:** 2026-05-21

**Authors:** Shakila Parveen Asrafali, Thirukumaran Periyasamy, Jaewoong Lee

**Affiliations:** Department of Fiber System Engineering, Yeungnam University, Gyeongsan 38541, Republic of Korea; shakilaparveen@yu.ac.kr (S.P.A.); thirukumaran@ynu.ac.kr (T.P.)

**Keywords:** electrochemical sensors, nanostructured materials, food safety, smart packaging, real-time monitoring, nanomaterials, metal oxides, MXene, graphene

## Abstract

Rising global demand for safe, high-quality foods has accelerated the development of rapid, sensitive, and cost-effective analytical technologies for detecting harmful substances and quality markers. Electrochemical sensors have emerged as promising tools for food safety monitoring due to their high sensitivity, fast response, portability, and affordability compared with conventional laboratory methods. This review highlights recent advances in nanostructured electrochemical sensors for detecting key food analytes, including antioxidants, mycotoxins, allergens, and flavor compounds in diverse food matrices. It examines advanced nanomaterials such as metal oxides, MXenes, doped carbon nitrides, and noble metal-decorated graphene, which enhance sensor performance through improved surface area, conductivity, and electrocatalytic activity. Integrated with screen-printed or glassy carbon electrodes, these materials achieve ultra-low detection limits, wide linear ranges, and strong selectivity in complex food systems. The review also explores next-generation applications such as NFC-enabled smart packaging for continuous, non-invasive monitoring across the supply chain. Emerging trends in miniaturization, multiplex sensing, and artificial intelligence are discussed, along with key challenges in translating laboratory innovations into practical commercial solutions for global food safety.

## 1. Introduction

### 1.1. The Global Food Safety Challenge and Traditional Analytical Limitations

Food safety represents one of the most pressing public health challenges of the 21st century, with the World Health Organization estimating that approximately 600 million people fall ill annually due to harmful substances in food, resulting in 420,000 deaths worldwide and creating an enormous burden on healthcare systems globally. As global food supply chains become increasingly complex and interconnected, spanning multiple countries and continents with numerous processing stages and distribution networks, the risk of harmful substances from biological pathogens, including bacteria, viruses, and parasites, chemical toxins such as mycotoxins and heavy metals, unauthorized additives and adulterants, and allergens that can trigger severe reactions in sensitive individuals has escalated dramatically [1,2,3,4,5]. The economic burden of foodborne illnesses encompasses not only direct healthcare costs for treating affected individuals but also productivity losses from illness and disability, trade disruptions when safety events affect international commerce, recall expenses that can bankrupt small producers, litigation costs, and damage to brand reputation, collectively amounting to billions of dollars annually across developed and developing nations. This multifaceted challenge, affecting public health, economic stability, and consumer confidence, underscores the critical need for robust, reliable, and accessible food safety monitoring technologies that can be deployed at multiple points throughout the food production and distribution system, from farm fields and processing facilities to distribution centers, retail locations, and ultimately consumer homes.

Traditional analytical methods for food safety testing, including high-performance liquid chromatography, which separates compounds based on differential partitioning between mobile and stationary phases; gas chromatography–mass spectrometry, which combines separation with molecular identification through mass-to-charge ratios; enzyme-linked immunosorbent assays, which use antibody–antigen interactions for detection; and microbiological culture techniques that grow and identify pathogenic organisms, while highly accurate, well-established in regulatory frameworks, and accepted as gold standards, face significant limitations that restrict their utility in modern food safety applications. These conventional approaches typically require expensive laboratory infrastructure with controlled environmental conditions, including temperature and humidity regulation; sophisticated instrumentation that demands regular calibration and maintenance and often costs hundreds of thousands of dollars; highly trained personnel with specialized expertise in analytical chemistry or microbiology; lengthy analysis times ranging from several hours to multiple days, particularly for microbiological methods; and extensive sample preparation protocols involving multiple steps of extraction, purification, concentration, and sometimes derivatization that consume reagents and introduce potential errors [6,7,8]. Furthermore, these methods are generally unsuitable for on-site, real-time monitoring applications due to their reliance on centralized laboratory facilities and bulky equipment, creating critical gaps in the food safety surveillance continuum from farm to fork. The inability to perform rapid screening at production facilities where immediate corrective actions could prevent unsafe products from entering the supply chain, at distribution centers where temperature abuse or package integrity failures might occur, at retail locations where storage conditions vary, and at consumer points of use where individuals could make informed decisions about consumption, means that safety issues may remain undetected until products have been widely distributed and consumed, leading to large-scale outbreaks affecting thousands of individuals and necessitating costly recalls.

### 1.2. Electrochemical Sensors as Transformative Analytical Platforms

**Figure 1:**
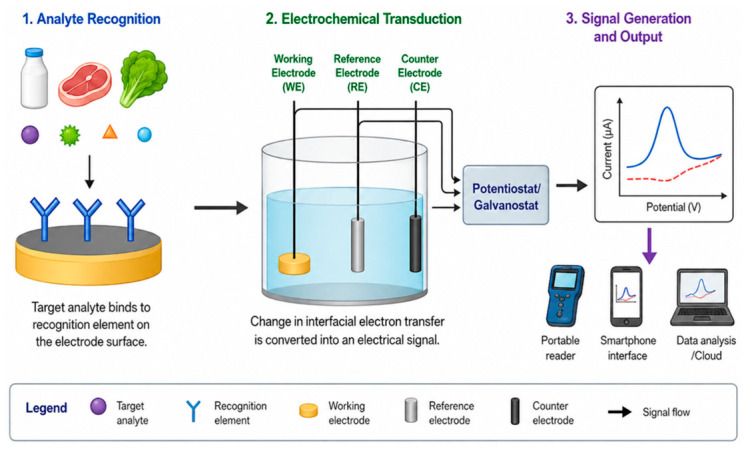
Schematic of an electrochemical sensor for food safety assessment.

Electrochemical sensors have emerged as transformative analytical platforms that address many of the fundamental limitations inherent in traditional food testing methods, offering a compelling alternative that combines high performance with practical advantages suited to modern food safety needs. These devices operate by transducing chemical or biochemical recognition events occurring at molecular scales into measurable electrical signals through redox reactions where electrons are transferred between analytes and electrodes, ion transfer processes where charged species move across interfaces, or capacitive changes where the electrical properties of electrode–solution interfaces are altered [9,10,11,12,13]. The fundamental principle underlying electrochemical sensing involves the direct conversion of chemical information into electronic signals without requiring complex optical systems, expensive separation columns, or extensive sample manipulation, thereby enabling simpler, faster, and more cost-effective analytical workflows that can be deployed outside traditional laboratory settings (Figure 1).

The high sensitivity and selectivity achievable with modern electrochemical sensors represent perhaps their most compelling advantage for food safety applications, as contemporary platforms employing advanced nanomaterials and optimized detection strategies can detect target analytes at ultra-trace levels ranging from picomolar to femtomolar concentrations, corresponding to parts per trillion or even parts per quadrillion in some cases, with excellent specificity that enables discrimination of target compounds even in complex food matrices containing thousands of potentially interfering compounds, including structurally similar molecules, electroactive species that undergo redox reactions at similar potentials, and matrix components that can adsorb to electrode surfaces or participate in side reactions. This exceptional sensitivity arises from multiple factors, including the direct nature of electrochemical transduction, where each molecular recognition event can potentially contribute to the measured signal without losses through intermediate conversion steps; the use of advanced nanomaterials that amplify signals through catalytic enhancement of electron transfer kinetics and dramatically increased surface areas, providing more sites for analyte interaction; and sophisticated detection techniques like differential pulse voltammetry that discriminate against background signals while preserving analytical information [14,15,16]. The selectivity of electrochemical sensors can be tailored through multiple strategies, including judicious choice of detection potential to exploit differences in redox potentials between target analytes and interferents; electrode surface chemistry modifications that favor specific interactions with target molecules through electrostatic effects, hydrophobic–hydrophilic balance, or coordination chemistry; and incorporation of molecular recognition elements such as antibodies that bind specifically to target analytes with dissociation constants in the nanomolar to picomolar range, aptamers that are synthetic oligonucleotides selected for specific binding, or molecularly imprinted polymers that create synthetic recognition sites complementary in shape and functionality to target molecules.

Rapid response times represent another critical advantage of electrochemical sensing platforms that distinguish them from conventional methods, with analysis times typically reduced to minutes or even seconds compared to the hours or days required by chromatographic separations, immunoassays with lengthy incubation steps, or microbiological cultures requiring overnight or multi-day growth periods [17,18]. This speed enables real-time or near-real-time monitoring applications that are critical for time-sensitive food safety decisions, such as screening incoming raw materials at processing facilities to prevent toxic substances from being incorporated into products, monitoring critical control points during production to ensure process parameters remain within safe ranges, verifying product quality before shipment to ensure only safe products reach consumers, and enabling consumers to assess food safety at the point of purchase or immediately before consumption to make informed decisions about whether products are safe to eat. The kinetic advantages of electrochemical detection stem from the inherently fast electron transfer processes occurring at electrode surfaces, which can reach equilibrium in milliseconds to seconds, and the elimination of time-consuming separation steps, lengthy incubation periods for antibody-antigen binding to reach equilibrium, or growth periods for microorganisms to reach detectable levels that are required by many traditional methods.

Cost-effectiveness provides a compelling economic rationale for electrochemical sensor adoption, which is particularly important in the food industry, where profit margins are often thin and testing costs must be minimized while maintaining adequate safety assurance. Electrochemical sensors require minimal reagents compared to chromatographic methods that consume expensive mobile phases and immunoassay methods that require costly antibodies and enzyme conjugates for each test. They can be mass-produced using established microfabrication techniques adapted from the electronics industry, including screen printing, photolithography, and thin-film deposition, which enable economies of scale and eliminate the need for expensive laboratory equipment, including chromatographs costing hundreds of thousands of dollars and requiring dedicated laboratory space with controlled environments. The per-test costs for electrochemical sensors, particularly when disposable electrode formats are employed, can be orders of magnitude lower than those for conventional methods, potentially below one dollar per test compared to five to fifty dollars for chromatographic or immunoassay methods, making comprehensive food safety testing economically feasible even for lower-value food products like grains, where testing costs must be kept extremely low, and enabling testing at scales previously impractical due to cost constraints, such as testing every production batch or even individual packages rather than relying on representative sampling.

The portability and miniaturization capabilities inherent to electrochemical sensing platforms represent transformative advantages for decentralized food safety monitoring, enabling testing to be performed where and when it is needed rather than requiring sample transport to centralized laboratories with attendant delays and potential sample degradation. The fundamental compatibility of electrochemical sensors with microelectronics and microfabrication technologies, leveraging decades of development in the semiconductor industry, enables development of portable, handheld, and even wearable sensing devices that can be deployed in field settings far from centralized laboratory facilities, including agricultural production areas where crops are harvested, food processing facilities where products are manufactured, distribution networks including warehouses and transportation vehicles where temperature abuse might occur, retail environments where consumers make purchasing decisions, and even consumer homes where individuals can verify safety before consumption [19,20,21,22]. Modern potentiostats, the electronic instruments that control electrode potentials and measure resulting currents to perform electrochemical measurements, have been miniaturized from rack-mounted laboratory instruments to handheld devices smaller than smartphones and even chip-scale implementations that can be integrated directly with sensors, enabling sophisticated electrochemical measurements to be performed anywhere with battery-powered or even energy-harvesting equipment. This portability is particularly valuable for monitoring food safety in resource-limited settings, including developing countries where centralized laboratory infrastructure is inadequate, remote agricultural regions far from cities where laboratories are located, and emergency situations where rapid deployment of testing capabilities is needed.

The versatility of electrochemical platforms in detecting diverse analyte classes, from small organic molecules with molecular weights below 500 daltons to large proteins exceeding 100,000 daltons, intact pathogens including bacteria and viruses, and complex toxins with varied chemical structures, through appropriate electrode modification and recognition element selection, represents a key advantage that distinguishes electrochemical sensing from methods optimized for specific compound classes. Unlike chromatographic methods that work well for volatile or semi-volatile organic compounds but struggle with large biomolecules, or immunoassays that excel for proteins but are less suitable for small molecules that are poorly immunogenic, electrochemical sensors can be adapted to virtually any target analyte that can be made to participate in electron transfer reactions, either directly through inherent electroactivity or indirectly through mediators or enzymatic reactions, or that can be recognized by biorecognition elements whose binding produces detectable changes in interfacial electrochemical properties such as charge transfer resistance or double-layer capacitance. This versatility enables development of comprehensive food safety monitoring systems addressing multiple analyte classes, including toxic substances, biological pathogens, allergens, and quality markers, using common instrumentation and operational principles, simplifying training, reducing equipment costs, and enabling integrated approaches to food safety management.

### 1.3. The Nanomaterials Revolution in Electrochemical Sensing

The performance of electrochemical sensors is fundamentally determined by the properties of the working electrode surface, where all key processes occur, including analyte adsorption from solution to electrode surface, molecular recognition through specific or non-specific interactions, electron transfer between analytes and electrodes that generates the analytical signal, and sometimes catalytic reactions that amplify signals or enable detection of non-electroactive species. The advent of nanomaterials science over the past two decades has revolutionized electrochemical sensor design by providing unprecedented control over electrode surface properties at the molecular and atomic scales, enabling rational engineering of surface chemistry that determines how analytes interact with electrodes, morphology that affects surface area and mass transport, electronic structure that governs electron transfer kinetics and catalytic activity, and mechanical properties that influence sensor robustness and stability [23,24,25,26]. Nanomaterials, defined as materials with at least one dimension in the nanometer scale range of 1 to 100 nm, exhibit unique physical and chemical properties that differ dramatically from their bulk counterparts due to quantum confinement effects, where electronic energy levels become discrete rather than continuous, high surface-to-volume ratios, where the fraction of atoms at surfaces rather than in bulk reaches 10 to 50 percent compared to less than 0.01 percent for bulk materials, and the dominance of surface atoms over bulk atoms in determining overall material behavior, including reactivity, electronic properties, and mechanical properties.

Enhanced surface area represents one of the most immediately impactful advantages provided by nanostructured electrode materials, as nanomaterials can provide dramatically increased electroactive surface areas compared to bulk materials, often by factors of 100 to 1000 or more depending on the specific nanomaterial morphology and size distribution. This increased surface area directly amplifies electrochemical signals by providing more sites for analyte adsorption and electron transfer, with the current response in many cases being directly proportional to the electroactive area, thereby improving sensitivity and lowering detection limits by the same factor as the area increases. The high surface area of nanomaterials also enables immobilization of greater quantities of biorecognition elements such as antibodies or enzymes in biosensor applications, with loading capacities often 10 to 100 times higher than those achievable on planar electrodes, further enhancing sensitivity by increasing the number of recognition events that can occur and extending the linear detection range to higher analyte concentrations before saturation of binding sites occurs. The relationship between nanomaterial size and surface area is dramatic, with a one-micrometer cube having a surface-to-volume ratio of 6 square micrometers per cubic micrometer. Dividing this cube into one thousand cubes of 100 nm each increases the total surface area to 60 square micrometers while maintaining the same total volume, and further division into 10-nanometer cubes increases the surface area to 600 square micrometers, demonstrating how nanostructuring can increase surface area by orders of magnitude [27,28,29,30].

Electrocatalytic activity exhibited by many nanomaterials represents another critical advantage for electrochemical sensing, as these materials can accelerate electron transfer kinetics for redox-active analytes, effectively lowering the activation energy barriers for oxidation or reduction reactions and thereby reducing the overpotentials required to drive these reactions to measurable rates and increasing current responses for a given analyte concentration. Electrocatalysis by nanomaterials can shift detection potentials to more favorable regions where fewer interfering compounds are electroactive, improving selectivity by enabling detection at potentials where background currents from matrix components are minimized, and can increase current responses by factors of 10 to 1000 compared to unmodified electrodes, improving sensitivity and lowering detection limits proportionally. The catalytic properties of nanomaterials arise from multiple factors, including their high densities of active sites, often located at edges, corners, defects, or specific crystal facets where atoms have lower coordination numbers and higher reactivity compared to smooth surfaces, and from their electronic structures that can facilitate electron transfer between electrodes and analytes by providing appropriate energy levels that match analyte redox potentials, reducing reorganization energies required for electron transfer, or providing multiple oxidation states that can shuttle electrons [31,32]. The mechanisms of electrocatalysis are diverse and can involve direct electron transfer, where analytes undergo redox reactions directly at catalytic sites on nanomaterial surfaces; mediated electron transfer, where the nanomaterial itself undergoes reversible redox reactions and shuttles electrons between the electrode and analytes; or chemical catalysis, where the nanomaterial catalyzes chemical reactions of analytes that produce electroactive products.

Tunable electronic properties represent a sophisticated advantage of nanomaterials that enables precise optimization of sensor performance through materials engineering at the atomic and molecular levels. Quantum confinement effects in nanoscale materials, where the physical dimensions become comparable to or smaller than the de Broglie wavelength of charge carriers, can modify electronic band structures by creating discrete energy levels rather than continuous bands, alter work functions that determine the energy required to remove electrons from materials, and change charge carrier mobilities that affect how rapidly electrons or holes can move through materials in response to electric fields. These electronic property modifications enable the matching of nanomaterial energy levels to analyte redox potentials to minimize overpotentials and maximize electron transfer rates, optimization of electron transfer rates by tuning the electronic coupling between nanomaterials and electrodes or between nanomaterials and analytes, and control of surface reactivity toward specific analytes by adjusting the energy of surface states that participate in adsorption and catalysis. The ability to tune electronic properties through nanomaterial engineering—by controlling particle size with smaller particles exhibiting stronger quantum confinement effects, adjusting composition through doping or alloying to introduce charge carriers or modify band structures, modifying surface chemistry through functionalization with organic or inorganic groups that can donate or accept electrons, and controlling crystal structure through synthesis conditions that favor specific phases with different electronic properties—provides a powerful design tool for creating sensors optimized for specific applications and analytes.

Multifunctionality achievable through hybrid and composite nanomaterials represents an advanced capability that enables integration of multiple desirable properties, including high electrical conductivity necessary for rapid electron transfer and low resistance, strong electrocatalytic activity for signal amplification and selectivity enhancement, biocompatibility for biosensor applications where proteins or cells must maintain activity and stability, and molecular recognition capabilities for specific analyte binding within single integrated platforms that can address the complex, often competing requirements of practical sensor applications. By combining different nanomaterial classes with complementary properties, such as highly conductive graphene that provides excellent electron transport and high surface area with catalytically active metal nanoparticles that accelerate specific redox reactions, or electrically active metal oxides that provide catalytic sites and tunable electronic properties with biocompatible polymers that prevent protein denaturation and improve processability, researchers can create synergistic systems whose performance exceeds what could be achieved with any single material through additive effects where properties simply add together or, more powerfully, through synergistic effects where new properties emerge from interactions between materials. This multifunctionality is particularly valuable for addressing the complex requirements of real-world food sensor applications, where high sensitivity to detect analytes at regulatory limits, good selectivity to discriminate targets from thousands of matrix components, adequate stability to maintain performance during storage and repeated use, and compatibility with complex sample matrices that contain proteins, lipids, carbohydrates, salts, and numerous other compounds must all be achieved simultaneously, often requiring careful balancing of competing factors [33,34,35].

Recent years have witnessed explosive growth in the development and application of diverse nanomaterials for food sensor applications, spanning multiple material classes with distinct advantages, synthesis methods, and optimal application domains. Metal oxide nanostructures, including binary oxides such as tin sulfide with its layered structure and narrow band gap and ternary oxides like nickel tungstate combining nickel’s electrochemical activity with tungsten’s stability, tungsten antimonate with unique electronic band structures, and calcium molybdate with its scheelite structure, offer high chemical stability across wide pH ranges from strongly acidic to strongly basic and temperature ranges from below freezing to above 100 °C, tunable band gaps that can be adjusted through composition, doping, and nanostructuring to match specific applications, and excellent electrocatalytic properties arising from multiple oxidation states of metal centers and surface oxygen species that can participate in redox reactions. Two-dimensional materials, including MXenes such as Ti_3_C_2_T_x_ with metallic conductivity and hydrophilic surfaces, graphene and its derivatives offering exceptional electrical properties and chemical versatility, and transition metal dichalcogenides like MoS_2_ and WS_2_ providing tunable band gaps and catalytic edge sites, provide exceptional electrical conductivity due to extended pi-electron systems in graphene or metallic bonding in MXenes. They also have extremely high aspect ratios with lateral dimensions in micrometers and thicknesses of single atomic layers maximizing interfacial contact with analytes and electrolytes, and unique surface chemistries that can be further functionalized for specific applications through covalent attachment of organic groups, coordination of metal ions, or intercalation of molecules between layers.

Carbon-based nanomaterials represent a particularly diverse and widely studied class, encompassing functionalized graphene with controlled introduction of oxygen, nitrogen, or other heteroatoms that modify electronic properties and provide sites for further functionalization. Carbon nanotubes offer high aspect ratios and excellent conductivity in one-dimensional structures, while carbon nitrides provides nitrogen-rich surfaces for coordination chemistry and metal-free catalysis. Numerous composites combine these materials with other functional components, including metal oxides, conducting polymers, or metal nanoparticles [36]. The carbon-based nanomaterials combine excellent electrical conductivity derived from sp^2^ hybridized carbon networks, where electrons are delocalized over extended pi systems, with remarkable chemical versatility arising from the ability to introduce diverse functional groups through oxidation, creating hydroxyl, carboxyl, and epoxide groups; reduction, removing oxygen functionalities; doping, substituting nitrogen, boron, sulfur, or phosphorus for carbon atoms; or covalent modification, attaching organic molecules through carbon–carbon or carbon–heteroatom bonds. Noble metal nanoparticles, including gold with its excellent biocompatibility and ease of functionalization; silver, offering antimicrobial properties and lower cost; and platinum, providing outstanding catalytic activity, particularly for hydrogen peroxide detection, offer outstanding catalytic activity for numerous redox reactions relevant to food safety applications including oxidation of phenolic compounds, amines, alcohols, and thiols and reduction of peroxides, nitro compounds, and oxygen. Additionally, they exhibit excellent biocompatibility, which is particularly important for biosensor applications where proteins must maintain their native structure and activity. Their well-established surface chemistry enables straightforward functionalization with thiols that form strong gold-sulfur bonds, amines that can coordinate to metal surfaces, or other ligands that enable attachment of recognition elements or protective coatings. Hybrid nanocomposites represent the frontier of nanomaterial development for electrochemical sensing, combining multiple material classes in carefully designed architectures that provide synergistic combinations of desirable properties optimized for specific sensing challenges, with examples including metal oxide–carbon composites that combine catalytic activity with conductivity and surface area, MXene-polymer hybrids that combine electrical properties with processability and mechanical flexibility, and multi-component systems with three or more distinct materials arranged in core-shell, layered, or interpenetrating network architectures [37,38,39]. Table 1 includes the advantages and disadvantages of carbon-based and non-carbon based nanomaterials used in electrochemical food sensors.

### 1.4. Smart Packaging

#### Integration and the Vision for Comprehensive Food Safety

Beyond laboratory-based sensing applications focused on quality control testing in centralized facilities and regulatory compliance verification, the integration of electrochemical sensors into intelligent food packaging represents a transformative approach to continuous food quality monitoring throughout the supply chain. This fundamentally changes how food safety is managed by shifting from periodic testing at discrete points to continuous assessment providing real-time information. Smart packaging technologies incorporating wireless-enabled sensors embedded directly in food packaging materials or attached to package surfaces can provide real-time information about food freshness through detection of metabolites indicating microbial growth or enzymatic degradation, spoilage progression through monitoring of volatile organic compounds or pH changes, safety status through detection of pathogens or toxins, and authenticity through verification of product identity and supply chain integrity. This information is delivered directly to consumers who can scan packages with smartphones, retailers who can optimize inventory management and reduce waste, and supply chain managers who can identify where quality issues occur and implement necessary actions [40,41]. This continuous monitoring capability addresses a critical gap in current food safety systems, which typically rely on date labels indicating maximum shelf-life under ideal storage conditions but cannot account for temperature effects during transportation or storage, package integrity failures that allow safety hazards or accelerate spoilage, or individual product variations in initial microbial loads or chemical composition that affect actual shelf-life.

Near-field communication (NFC) technology has emerged as an ideal platform for sensor-integrated packaging due to several compelling advantages over alternative wireless technologies. NFC’s widespread adoption in smartphones, with over 2 billion NFC-enabled devices globally, means that consumers already have the necessary reader hardware without requiring dedicated equipment purchases. This enables immediate deployment without infrastructure investment. Low power requirements enabling passive operation, where tags are powered entirely by electromagnetic fields generated by readers, eliminate the need for batteries that would add cost—typically several dollars per battery—bulk that would increase package size, weight that would increase shipping costs, and environmental concerns from battery disposal. This approach also potentially limits shelf-life through battery depletion over months or years. Secure data transmission protocols built into NFC standards prevent tampering with sensor data and ensure data integrity through encryption and authentication, which is critical for applications where food safety decisions depend on sensor readings. Established manufacturing infrastructure that can be adapted for sensor integration leverages existing NFC tag production facilities and supply chains, reducing the capital investment required to scale up production and accelerating time to market [9,10].

**Figure 2:**
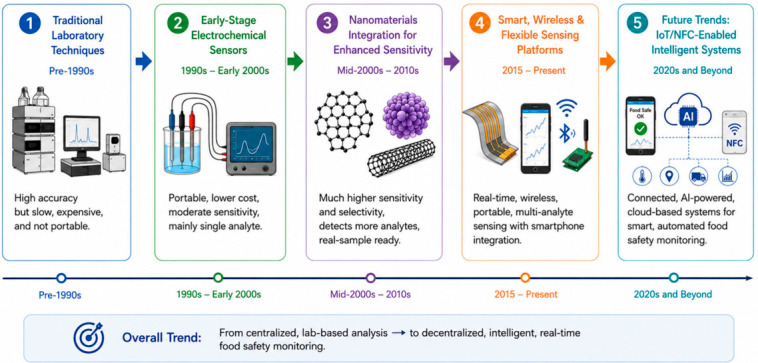
Development progress chart of food sensing technologies.

The convergence of advanced nanomaterials providing sensitive and selective detection capabilities through optimized surface chemistry and electronic properties, electrochemical sensing platforms offering simple, low-power operation compatible with energy harvesting from NFC fields, wireless communication technologies enabling seamless data transmission without requiring physical connections or complex pairing procedures, and data analytics including artificial intelligence and machine learning for predictive modeling that can forecast remaining shelf-life based on sensor time-series data accounting for actual storage conditions rather than assuming ideal conditions, is creating unprecedented opportunities for comprehensive food safety management systems (Figure 2). These integrated systems can detect safety issues at early stages before they become health hazards by identifying metabolites or other markers of pathogen growth when microbial populations are still low, predict remaining shelf-life with greater accuracy than simple date labels by continuously monitoring quality changes and using mathematical models to extrapolate future degradation, authenticate products and verify supply chain integrity to combat counterfeiting and fraud that costs the food industry billions of dollars annually, and provide complete traceability from production to consumption, enabling rapid response to safety incidents by quickly identifying affected products and their distribution [40,41,42,43,44,45]. The realization of this vision requires continued innovation not only in sensor materials and designs but also in system integration, bringing together sensors, power management, data processing, and communication in compact, robust packages, data management infrastructure including cloud platforms for aggregating data from millions of packages, regulatory frameworks that establish standards for sensor performance and data reporting, and business models that can support widespread deployment of smart packaging technologies by demonstrating clear return on investment through waste reduction, quality assurance, or premium pricing.

## 2. Materials and Methods

In this study, a generative artificial intelligence (GenAI) tool was used solely for the preparation of Figure 1 and Figure 2 to improve visual presentation and scientific communication. The keywords ‘Electrochemical sensor for food safety assessment’ and ‘Development progress chart of food sensing technologies’ were used to generate Figure 1 and Figure 2, respectively. The AI-assisted images were reviewed, edited, and validated by the authors to ensure their accuracy, originality, and relevance to the study.

## 3. Fundamentals of Electrochemical Sensing

Electrochemical sensors operate based on fundamental electrochemical principles that govern charge transfer processes occurring at electrode-electrolyte interfaces, where the boundary between the solid electrode material and the liquid solution phase creates a complex interfacial region characterized by electric fields arising from charge separation, charge distributions where ions accumulate or deplete near surfaces, and concentration gradients where analyte concentrations near electrodes differ from the bulk solution due to consumption or production by electrode reactions. The three primary electrochemical detection modes employed in food safety applications each offer distinct advantages and are selected based on the specific requirements of the analytical problem, including the nature of the target analyte and whether it is inherently electroactive or requires indirect detection, the complexity of the sample matrix, the level of interfering compounds, and the desired performance characteristics, including detection limits, analysis speed, and selectivity.

Voltammetric techniques involve applying a controlled potential to the working electrode while measuring the resulting current that flows through analytes undergoing oxidation or reduction reactions at the electrode surface, with the potential typically varied over time according to specific waveforms that optimize sensitivity and selectivity. The relationship between the applied potential and the measured current provides both qualitative information through the potential at which current peaks occur, which is characteristic of specific analytes and their redox chemistry and can be used for identification, and quantitative information through the magnitude of peak currents, which is proportional to analyte concentration according to well-established electrochemical theories, including the Randles–Sevcik equation for reversible reactions and the Cottrell equation for diffusion-controlled processes. Cyclic voltammetry represents a widely used technique primarily for mechanistic studies and electrode characterization rather than routine analytical measurements, involving sweeping the potential linearly from an initial value to a switching potential and then back to the initial value while recording current. The resulting current–potential plot, called a cyclic voltammogram, provides rich information about redox potentials indicating the thermodynamic favorability of electron transfer reactions, the reversibility of redox processes which affects sensor reusability and whether analytes can be regenerated after detection, and electron transfer kinetics that determine sensor response speed and the overpotentials required to drive reactions at measurable rates [46,47,48,49,50]. Differential pulse voltammetry represents a highly sensitive technique that applies a series of potential pulses superimposed on a linear potential ramp and measures current differences before and after each pulse, effectively discriminating against capacitive background currents that arise from the charging of the electrode–solution interface and do not contain analytical information while preserving faradaic currents arising from analyte redox reactions that carry the analytical signal. This discrimination dramatically improves signal-to-noise ratios by factors of 10 to 100 compared to simpler techniques and enables detection of analytes at much lower concentrations than possible with linear sweep voltammetry or even cyclic voltammetry, with detection limits often in the nanomolar to picomolar range for optimized systems.

Amperometric detection operates by applying a constant potential to the working electrode and measuring the resulting steady-state current, which is proportional to analyte concentration according to the Cottrell equation for diffusion-controlled processes or to analyte flux for convective systems. This technique offers excellent sensitivity due to the absence of capacitive charging currents that decay rapidly after potential application, leaving only faradaic currents from analyte reactions, a wide linear dynamic range spanning multiple orders of magnitude in concentration from nanomolar to millimolar levels in many cases, and compatibility with flow systems and sensor arrays, enabling multiplexed detection of multiple analytes simultaneously or continuous monitoring applications where samples flow continuously past electrodes. Amperometric detection is particularly well-suited for biosensor applications where enzymes catalyze reactions producing electroactive products such as hydrogen peroxide from oxidase enzymes or NADH from dehydrogenase enzymes [45,49], which can be detected amperometrically at appropriate potentials with high sensitivity.

Electrochemical impedance spectroscopy represents a fundamentally different approach that measures the impedance of an electrochemical system, which characterizes its opposition to alternating current flow, over a range of frequencies typically spanning from millihertz to megahertz. It provides a frequency spectrum that can be analyzed to extract information about different processes occurring at different timescales. The impedance measurements provide information about interfacial properties, including double-layer capacitance that reflects the structure and composition of the electrode–solution interface and how it changes upon analyte binding, charge transfer resistance that quantifies the kinetic barrier to electron transfer reactions and increases when electrode surfaces are blocked by non-conductive species, and mass transport phenomena, including diffusion of analytes through solution or through surface films that determine how rapidly analytes can reach electrode surfaces. EIS is particularly valuable for biosensor applications where binding events between biorecognition elements like antibodies and target analytes alter interfacial properties without necessarily involving direct electron transfer, providing a label-free detection mechanism that does not require modification of analytes with electroactive or fluorescent tags. The binding of proteins to antibodies immobilized on electrode surfaces, for example, increases charge transfer resistance by partially blocking the electrode surface and increasing the distance between electrons and redox probes in solution, with the magnitude of resistance increase being proportional to the amount of protein bound and thus to analyte concentration [33,36].

A typical three-electrode electrochemical sensor system comprises three distinct electrodes, each serving a specific function in the measurement circuit, arranged in an electrochemical cell containing the sample solution and connected to a potentiostat that controls potentials and measures currents with high precision. The working electrode represents the primary sensing element where target analyte recognition and signal transduction occur through carefully engineered surface chemistry determining how analytes interact with the electrode, morphology affecting surface area and mass transport, and composition providing catalytic activity and electronic properties. The reference electrode provides a stable, known potential reference against which the working electrode potential is controlled, ensuring reproducible electrochemical conditions across measurements and enabling comparison of results between different laboratories and instruments using the same reference system. The counter electrode completes the electrical circuit and carries current flow without participating in the sensing reaction, serving to balance the current generated at the working electrode without introducing additional electrochemical processes that could interfere with measurements or consume analytes.

Modern sensor designs increasingly employ screen-printed electrode platforms that integrate all three electrodes onto single disposable substrates, typically ceramic or polymer materials, using thick-film printing techniques adapted from electronics manufacturing [19,20,21,22]. These integrated electrode systems offer numerous advantages, including low manufacturing costs through high-volume production using automated printing equipment, excellent reproducibility of electrode geometry and composition across production batches due to the controlled nature of printing processes, compact designs requiring minimal sample volumes—often less than 50 microliters—enabling testing of small samples or precious samples where larger volumes are unavailable, elimination of product-to-product transfer through a single-use disposable format where each measurement uses a fresh electrode, and simplified operation suitable for non-expert users in field settings without requiring extensive training in electrochemical techniques.

## 4. Advanced Nanomaterials for Food Sensor Applications

The selection and engineering of electrode materials represent the most critical factor determining electrochemical sensor performance, as the electrode surface is the place where all key processes occur, including analyte adsorption from solution, recognition through specific or non-specific interactions, electron transfer generating the analytical signal, and catalytic reactions that can amplify signals or enable detection of non-electroactive species. Metal oxides constitute a major class of electrode materials, offering exceptional chemical stability across wide ranges of pH (from strongly acidic to strongly basic), temperature (from below freezing to above 100 °C), and redox conditions. Furthermore, their electronic properties can be tuned through composition variation by mixing different metals and doping strategies to introduce foreign atoms into crystal lattices, and diverse morphologies can be achieved through various synthesis methods, including hydrothermal, sol–gel, precipitation, and electrodeposition techniques. Their wide band gaps, ranging from 1 to 6 eV, enabling semiconducting behavior, variable oxidation states that can participate in redox reactions and shuttle electrons, and oxygen-rich surface chemistry that can interact with organic analytes through hydrogen bonding, electrostatic interactions, and coordination, make them particularly attractive for electrocatalytic applications in food sensing, where they can accelerate electron transfer for numerous analytes.

**Figure 3:**
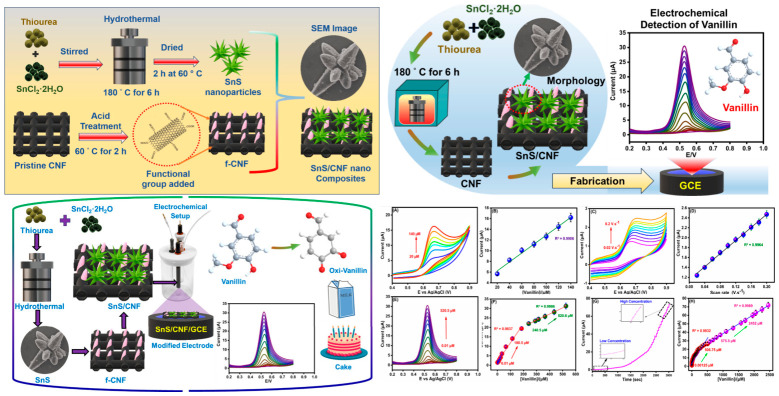
Schematic of SnS/CNF nanocomposite synthesis and its electrochemical sensing of vanillin. (**A**,**B**) CV response and calibration; (**C**,**D**) scan rate studies; (**E**,**F**) DPV response and linear plot; (**G**,**H**) amperometric analysis and corresponding calibration. Reproduced with permission from [25].

Binary metal oxides represent the simplest oxide systems, consisting of one metal element combined with oxygen in various stoichiometric ratios and crystal structures that determine properties. Tin sulfide (SnS), despite being a sulfide rather than an oxide, is often grouped with metal oxides due to similar properties, synthesis methods, and applications in electrochemical sensors [25]. SnS is a p-type semiconductor with a narrow band gap of around 1.3 eV, enabling visible light absorption, and a layered crystal structure similar to black phosphorus, with individual layers held together by weak van der Waals forces that can be disrupted to create high surface area nanostructures. This layered structure facilitates ion intercalation between layers during electrochemical reactions and provides high surface areas when synthesized as nanostructures through exfoliation or direct synthesis of thin nanosheets [27,28,29,30,31,32]. SnS nanoflowers synthesized through hydrothermal methods, where precursors react in aqueous solution at elevated temperatures around 150 to 200 °C in sealed vessels that generate autogenous pressure, exhibit hierarchical morphologies consisting of nanosheets assembled into flower-like three-dimensional structures with diameters of several micrometers (Figure 3). These hierarchical morphologies provide high surface areas through the nanoscale dimensions of individual nanosheet building blocks, which are tens of nanometers thick, and abundant edge sites that serve as active centers for electron transfer reactions due to their high surface energy and unsaturated coordination of surface atoms. When integrated into electrochemical sensors for tert-butylhydroquinone (TBHQ) detection, SnS-modified electrodes demonstrate ultra-low detection limits of 0.008 µM, corresponding to 0.0013 mg/L. This value falls well below regulatory limits even after accounting for sample dilution during analysis. Furthermore, the sensors exhibit wide linear ranges spanning from 0.01 to 919.7 µM, covering nearly five orders of magnitude [33,34,35,36] and enabling analysis of samples with widely varying TBHQ levels without requiring dilution or preconcentration steps. Additionally, the modified electrodes display excellent electrocatalytic activity toward TBHQ oxidation, which reduces overpotentials by several hundred millivolts compared to unmodified carbon electrodes and increases current responses by factors of 50 to 100. This provides good stability and reproducibility in complex edible oil samples containing numerous interfering compounds, including other phenolic antioxidants, tocopherols, and lipid oxidation products [37,38,39].

**Figure 4:**
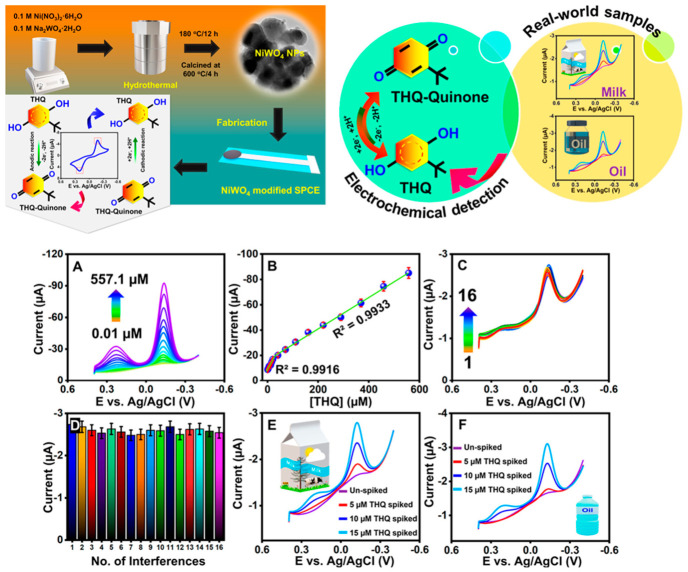
Schematic of hydrothermal synthesis and assembly of NiWO_4_/SPCE for THQ detection. (**A**,**B**) DPV response and calibration curve for THQ; (**C**,**D**) interference study and corresponding bar graph; (**E**,**F**) analysis of milk and edible oil samples. Reproduced with permission from [23].

Ternary metal oxides, consisting of two different metal elements combined with oxygen in specific stoichiometric ratios, offer additional degrees of freedom for property tuning through composition variation and often exhibit synergistic properties exceeding those of constituent binary oxides due to electronic interactions between different metal centers or structural effects arising from mixing metals with different ionic radii. Nickel tungstate, NiWO_4_, crystallizes in the monoclinic wolframite structure, where nickel and tungsten cations occupy octahedral sites coordinated by six oxygen atoms in a distorted close-packed oxygen lattice, creating chains of edge-sharing octahedra. This structure combines the electrochemical activity of nickel, which can undergo facile oxidation–reduction between Ni^2+^ and Ni^3+^ states, providing redox-active sites, with the chemical stability and electronic properties of tungsten oxides, which have wide band gaps of around 2.8 eV and can exist in multiple oxidation states from W^4+^ to W^6+^, enabling electron transfer. NiWO_4_ nanomaterials synthesized via hydrothermal methods [23], where metal salt precursors react in basic aqueous solutions at temperatures around 180 °C for 12 to 24 h, or co-precipitation methods, where metal salts are precipitated simultaneously from solution by adjusting pH followed by thermal treatment at 400 to 600 °C to crystallize the wolframite phase, exhibit various morphologies, including spherical nanoparticles with diameters of 20 to 100 nm, one-dimensional nanorods with diameters of 50 to 200 nm and lengths of several µm, and hierarchical nanoflowers assembled from nanosheet building blocks, depending on synthesis conditions, including precursor concentrations, temperature, time, and additives that control nucleation and growth (Figure 4). These nanostructures demonstrate high electrical conductivity due to the mixed valence states of nickel and tungsten, enabling charge carrier hopping between adjacent metal centers; excellent chemical stability across wide pH ranges from acidic to basic environments due to the strong metal–oxygen bonds in the wolframite structure; strong electrocatalytic activity for organic molecule oxidation arising from multiple redox-active sites and favorable electronic structure; and large surface areas when synthesized as nanoparticles or one-dimensional nanostructures, providing abundant sites for analyte adsorption and electron transfer.

**Figure 5:**
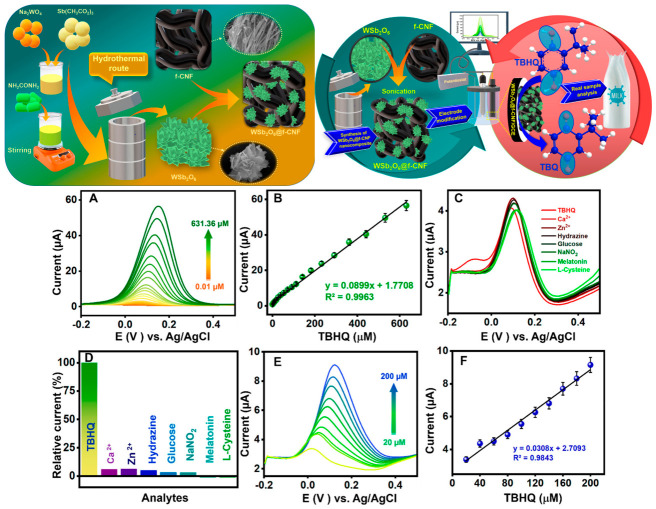
Schematic of WSb_2_O_6_/f-CNF composite synthesis via a hydrothermal–sonochemical route. (**A**,**B**) DPV response and calibration for TBHQ; (**C**,**D**) interference study and current comparison; (**E**,**F**) TBHQ detection in milk with a linear plot. Reproduced with permission from [24].

The effectiveness of tungsten antimonate nanoflowers (WSb_2_O_6_) anchored on functionalized carbon nanofibers (f-CNF) for TBHQ detection in milk was demonstrated [24]. WSb_2_O_6_ nanoflowers were synthesized using a hydrothermal approach. Aqueous solutions of antimony acetate and sodium tungstate were prepared, and polyvinylpyrrolidone was added as a surfactant. Urea was included to assist with morphological control. This mixture was transferred to a Teflon-lined autoclave and treated at 180 °C for 24 h. The resulting precipitate was washed and dried. Acid-functionalized carbon nanofibers (f-CNF) were prepared separately and combined with the WSb_2_O_6_ to form a nanocomposite. The resulting composite, WSb_2_O_6_/f-CNF electrode, exhibited exceptional electrocatalytic activity, antifouling behavior, and stability over repeated measurements. TEM micrographs reveal that WSb_2_O_6_ nanoflowers were irregularly distributed and self-aligned on the functionalized carbon nanofibers (f-CNF), indicating strong electrostatic interactions between the oxygen-rich carbon framework and WSb_2_O_6_, which promote their striped aggregation. Moreover, the distinct morphologies of both WSb_2_O_6_ and f-CNF were preserved in the final composite, suggesting that the individual structural advantages of each component were maintained, contributing to enhanced electrochemical performance. Their sensor had a linear detection range from 0.01 to 631 μM and it maintained accuracy during real-sample analysis in milk. The heterojunction formed between WSb_2_O_6_ and f-CNFs promoted superior electrocatalytic behavior, confirmed by their sensor’s limit of detection of 9 nM and a sensitivity of 0.127 μA·μM^−1^·cm^−2^. As shown in Figure 5, the unique nanoflower morphology, with abundant edge sites, ensured high surface-to-volume ratios, enhancing the interface for electron transfer and analyte interaction. Such architectural control over material structure plays a pivotal role in optimizing sensor performance.

To evaluate the selectivity of the WSb_2_O_6_/f-CNF/GCE-based sensor, various potential interfering species, including calcium ions (Ca^2+^), zinc ions (Zn^2+^), hydrazine, glucose, sodium nitrite (NaNO_2_), melatonin, and L-cysteine, were introduced into the electrolyte solution alongside TBHQ and analyzed using differential pulse voltammetry (DPV). The resulting relative current changes caused by these interferents were negligible, confirming that the proposed sensor exhibits high selectivity and sensitivity toward TBHQ detection. Its robust stability during repeated testing and ability to detect THQ in milk reinforce its value for continuous quality assurance in dairy processing. The limit of detection for TBHQ using the WSb_2_O_6_/f-CNF modified electrode, calculated via the standard deviation method, was determined to be 11 nM in a milk matrix. These results demonstrate that the developed electrochemical sensing platform is both reliable and effective for TBHQ detection in real-world samples, as evidenced by the high recovery rates [19,20,21,22,23,24,25,26]. Although the WSb_2_O_6_/f-CNF/GCE sensor showed effective TBHQ detection in milk with good recovery, the effects of major food-matrix components such as proteins, lipids, and carbohydrates were not fully examined. In milk, proteins may adsorb onto the electrode surface and cause fouling, lipids can form insulating layers that hinder electron transfer, and carbohydrates such as lactose may affect viscosity and analyte diffusion. The selectivity study mainly focused on small ions and molecules rather than the dominant macromolecules naturally present in milk. Therefore, further studies using real and model dairy matrices are needed to confirm sensor robustness and reduce the risks of false positive or false negative responses caused by matrix effects.

**Figure 6:**
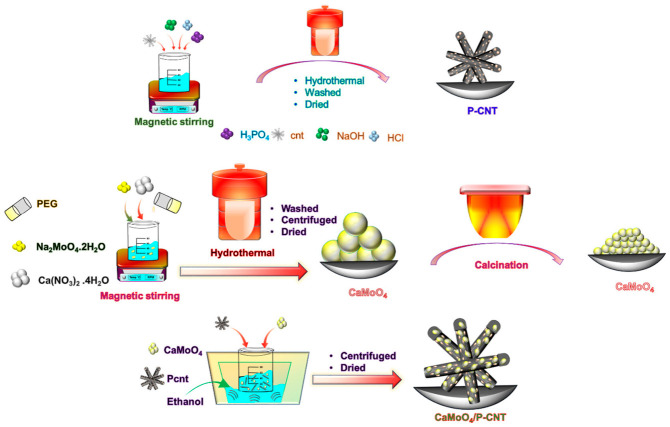

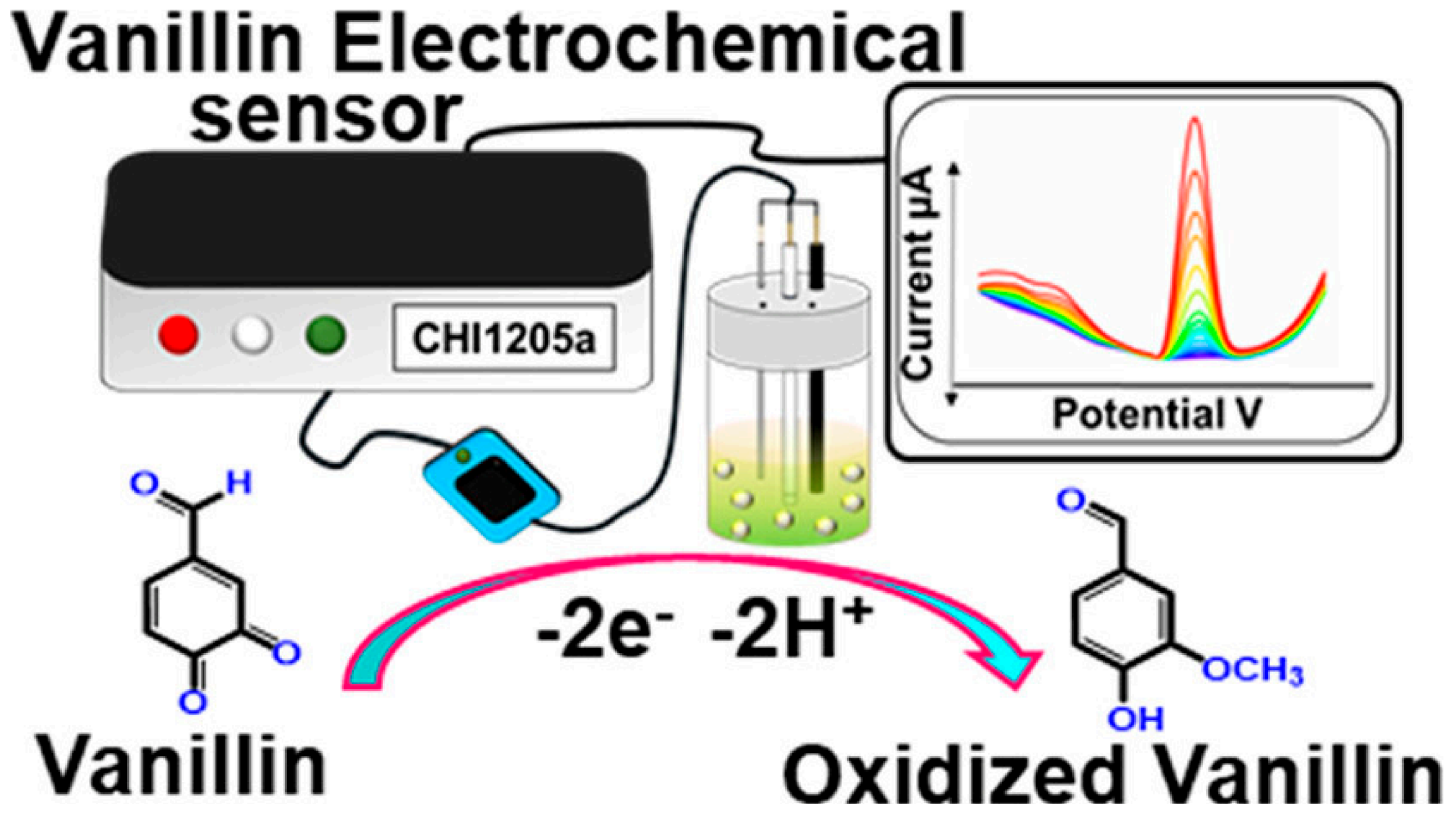
Scheme of CaMoO_4_/P-CNT nanocomposite synthesis and schematic of electrochemical vanillin detection. Reproduced with permission from [40].

Likewise, tetrahedrally structured CaMoO_4_ nanospheres grown on phosphorus-doped carbon nanotubes (P-CNTs) were synthesized [40] for the electrochemical detection of vanillin. At first, carbon nanotubes were doped with phosphorus through hydrothermal treatment with H_3_PO_4_ at 170 °C. CaMoO_4_ nanospheres were then synthesized and combined with P-CNTs using a sonochemical method, which used high-intensity ultrasonic waves to control morphology and ensure uniform coating. The resulting CaMoO_4_/P-CNT composite exhibited a high surface area, enhanced dispersion, and excellent electrochemical properties. The tetrahedral structure of CaMoO_4_ provided structural uniformity, while P-doping enhanced the CNTs’ electron-donating ability, as depicted in Figure 6. The combination of the electroactive CaMoO_4_ and the conductive CNT matrix facilitated a high electron transfer rate and broad analyte coverage. The efficient electrochemical detection of vanillin is largely attributed to the strong electrostatic interaction between P-CNT and CaMoO_4_. The enhanced current response observed during vanillin sensing is further supported by the synergistic effect between CaMoO_4_ and the P-CNT nanocomposite. This sensor demonstrated exceptionally low detection limits (DPV: 0.0017 μM; Amperometry: 0.00084 μM) and sensitivity values exceeding 1.9 μA μM^−1^ cm^−2^, suitable for high-throughput vanillin screening.

This performance is driven by multiple contributing factors, including the large electrochemical active surface area of the composite (0.15 cm^2^), the strong electrostatic interaction between CaMoO_4_ and P-CNT, and their synergistic effect. These features collectively enhance the interface between the electrode and electrolyte [41,42,43,44]. Specifically, CaMoO_4_ contributes to high electrocatalytic activity, while P-CNT offers an extended surface area for efficient vanillin ion adsorption. As a result, the CaMoO_4_/P-CNT/GCE electrode demonstrates superior sensitivity and effectiveness in vanillin sensing applications [45,46,47,48,49]. To assess the selectivity of the CaMoO_4_/P-CNT/GCE/RRDE electrocatalyst, differential pulse voltammetry (DPV) and amperometric techniques were employed in the presence of common interfering species. These included 50 μM concentrations of biomolecules such as glucose (Glu), ascorbic acid (AA), baking powder (BP), milk powder (MP), p-hydroxybenzoic acid (HBA), vanillic acid (VAA), and caffeine (CAF), as well as 100 μM concentrations of common ions like SO_4_^2−^, NO_3_^−^, and K^+^. The results indicate that the CaMoO_4_/P-CNT composite exhibits high selectivity and sensitivity toward vanillin, with no significant shift in its oxidation potential upon the addition of interferents, confirming the electrode’s robust performance in complex matrices. Its excellent repeatability and specificity in ice cream samples further affirm its relevance in routine food testing laboratories.

**Figure 7:**
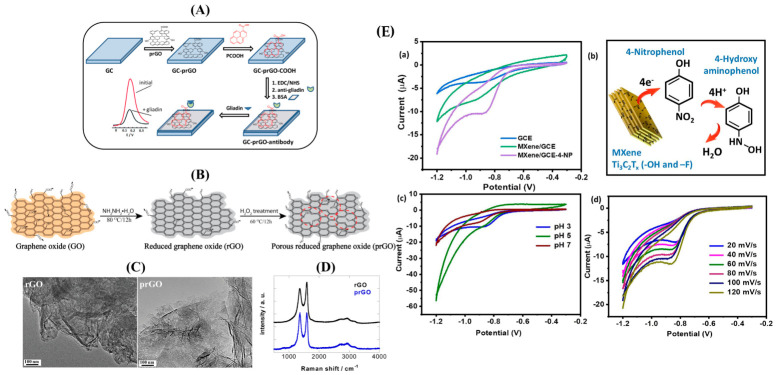
(**A**) Concept of label-free electrochemical gliadin immunosensor; (**B**) synthesis of prGO; (**C**) TEM images of rGO and prGO; (**D**) Raman spectra of rGO and prGO. (**E**) (**a**) CVs of bare and MXene/GCE with/without 4-NP; (**b**) schematic of 4-NP reduction on MXene; (**c**) CVs at different pH; (**d**) CVs at varying scan rates. Reproduced with permission from [26,56].

MXenes represent a revolutionary class of two-dimensional transition metal carbides, nitrides, and carbonitrides discovered in 2011 by Gogotsi and colleagues at Drexel University through selective etching of MAX phase precursors, opening a new field in two-dimensional materials beyond graphene. The name MXene derives from the precursor MAX phases, where M is an early transition metal like titanium, vanadium, niobium, or molybdenum; A represents an A-group element like aluminum, silicon, or gallium that is selectively removed during synthesis; and X is carbon or nitrogen forming strong bonds with the transition metal, with the -ene suffix indicating the two-dimensional layered structure analogous to graphene and distinguishing MXenes from their three-dimensional MAX phase precursors. The most extensively studied member, Ti_3_C_2_T_x_, where T_x_ represents surface terminations such as hydroxyl, oxygen, or fluorine groups introduced during the etching process, exhibits exceptional properties for electrochemical sensing that have driven rapid adoption in diverse applications, including energy storage, catalysis, sensing, and electromagnetic shielding. The structural characteristics of Ti_3_C_2_T_x_ include an atomically thin layered structure with a thickness of individual layers around 1 nm corresponding to three titanium atomic layers sandwiching two carbon atomic layers plus surface termination groups; a high aspect ratio with lateral dimensions reaching several micrometers while thickness remains nanoscale, creating extremely high surface-to-volume ratios; an accordion-like morphology with accessible interlayer spacing of approximately 1 nm that can accommodate ions, molecules, and even nanoparticles; and tunable surface chemistry through control of termination groups during synthesis by varying etchant composition or post-synthesis modification through chemical treatments or thermal annealing (Figure 7E).

MXene-modified electrodes for gliadin detection demonstrate exceptional performance with detection limits of 0.1 ng/mL, corresponding to approximately 2 ppm gluten in food extracts after accounting for extraction efficiency and dilution factors. The detection limit is well below the 20 ppm threshold defining gluten-free status, providing an adequate safety margin to account for measurement uncertainty. The electrodes exhibit linear ranges from 0.5 to 100 ng/mL, covering regulatory-relevant concentrations from well below to well above the gluten-free threshold. This enables both verification of gluten-free claims and quantification of gluten in affected products. Also, the method allows rapid detection within 10 min, including antibody incubation time and electrochemical measurement, representing a 10- to 20-fold time saving compared to the ELISA method, which requires 2 to 4 h for completion [26,51,52,53,54,55]. The electrodes show high selectivity against other food proteins, including corn zein from maize, rice glutelin from rice, and oat proteins from oats. Such selectivity minimizes cross-reactivity with proteins that might be present in gluten-free products or interfere in less selective assays. Excellent reproducibility is achieved, with relative standard deviations below 5% for intra-electrode measurements using the same electrode multiple times and 8% for inter-electrode measurements using different electrodes from the same manufacturing batch, indicating excellent control over MXene synthesis, electrode modification, and antibody immobilization procedures.

Carbon-based nanomaterials combine excellent electrical conductivity with remarkable chemical versatility, enabling diverse applications. Graphene, a single layer of sp^2^-hybridized carbon atoms arranged in a hexagonal lattice with carbon–carbon bond lengths of 0.142 nm, represents the archetypal two-dimensional material with extraordinary properties. Graphene nanoribbons, narrow strips of graphene with widths below 50 nm, exhibit quantum confinement effects that open band gaps absent in extended graphene sheets, creating semiconducting properties tunable through ribbon width and edge structure. Gold nanoparticle-decorated graphene nanoribbons combine the high conductivity and surface area of graphene with the electrocatalytic activity of gold nanoparticles in synergistic hybrid structures. Applications in diphenylamine (DPA) detection show picomolar detection limits from 0.001 to 0.01 µM, among the lowest reported for electrochemical DPA sensors and well below regulatory limits, wide linear ranges from 0.01 to 100 µM covering regulatory limits and typical residue levels found in treated fruits, rapid response times under 3 min enabling high-throughput screening of multiple samples per hour, excellent selectivity with selectivity coefficients exceeding 100 vs. common interferents, meaning the sensor responds 100 times more strongly to DPA than to interfering compounds, and high recovery in fruit samples from 98.2 to 102.5% demonstrating accuracy in complex matrices.

On the other hand, the detection of gliadin, the immunogenic fraction of gluten responsible for triggering celiac disease, was addressed [56] employing porous reduced graphene oxide (prGO) functionalized with anti-gliadin antibodies (Figure 7A,D). prGO was synthesized by oxidizing rGO with hydrogen peroxide, creating hydroxyl and epoxy groups that formed pores. The synthesized prGO was drop-cast on glassy carbon electrodes and modified using 1-pyrenecarboxylic acid as a linker. Antibodies were covalently immobilized using EDC/NHS chemistry. The final immunosensor was able to detect gliadin with high selectivity and sensitivity, crucial for gluten monitoring in food products for celiac patients. TEM analysis showed that rGO forms transparent nanosheets, while H_2_O_2_ treatment introduced uniform nanopores (4–6 nm), as shown in Figure 7C. Raman spectroscopy indicated increased defects in prGO, with the I_D_/I_G_ ratio rising from ~0.90 to ~0.98. Carboxylic groups were added using 1-pyrenecarboxylic acid (PCOOH), which attaches via π–π stacking without disrupting prGO’s conductivity, while improving wettability and enabling further modification.

The porous rGO surface facilitated efficient antibody immobilization and rapid electron transport. The resulting sensor achieved nanomolar sensitivity levels, achieving a low LoD of 1.2 ng/mL, with a linear range of 1.2–34 ng/mL for gliadin, a key gluten protein [56,57,58,59,60]. The biosensor showed excellent stability, with only 5% signal loss after 2 months at 4 °C, and reproducibility of 5.2% RSD at 20 ng mL^−1^ gliadin. It exhibited strong selectivity against common protein interferents and successfully detected gliadin in commercial food products, distinguishing between gluten-free and gluten-containing items, confirming its applicability for real-world food testing. Additionally, the sensor was successfully tested in real food samples, making it ideal for monitoring gluten-free products in the food industry. This work illustrates the use of carbonaceous nanomaterials, which can support both sensitivity and selectivity in biosensor configurations, where the sensor’s regenerability, speed and portability make it ideal for allergy-sensitive food labeling.

Graphitic carbon nitride with the formula g-C_3_N_4_ represents a polymeric semiconductor with a graphite-like layered structure composed of tri-s-triazine units connected through nitrogen bridges. Fluorine and phosphorus co-doped g-C_3_N_4_ exhibits synergistic enhancements, where dual-element doping creates effects exceeding what single-element doping achieves, with fluorine doping increasing conductivity and phosphorus doping creating additional active sites [41]. Sensors for TBHQ detection demonstrate ultra-low detection limits at sub-nanomolar levels, representing some of the lowest reported for any TBHQ sensor, wide linear ranges spanning 4 to 5 orders of magnitude from 0.001 to 100 µM covering regulatory limits and typical use levels, excellent selectivity in edible oil matrices, and robust stability with greater than 90% response retention after 30 days [42,43,44,45].

Conducting polymers like polypyrrole offer good electrical conductivity, biocompatibility, and ease of synthesis [61]. Hierarchical metal oxide–polypyrrole nanocomposites exhibit enhanced properties through the synergistic combination of oxide catalytic activity and polymer conductivity. These nanocomposites demonstrate exceptional performance in detecting diphenylamine with detection limits below 10 nanomolar, linear ranges exceeding three orders of magnitude, excellent reproducibility with relative standard deviations below 3%, high recovery from 98 to 102%, and long-term stability [61,62,63,64,65]. Noble metal nanoparticles, including gold, exhibit excellent electrocatalytic activity, biocompatibility, and ease of surface functionalization. Integration with carbon nanomaterials creates hybrid platforms combining high surface area, catalytic activity, enhanced electron transfer, and improved dispersion, preventing nanoparticle aggregation that would reduce performance. Table 2 includes the comprehensive critical summary of nanomaterials for electrochemical food sensing.

Across the discussed nanomaterials, no single class simultaneously satisfies the competing requirements of sensitivity, selectivity, stability, and scalability, making material choice inherently application-dependent. Metal oxides (e.g., NiWO_4_, WSb_2_O_6_, CaMoO_4_) offer strong chemical stability and tunable redox activity, but suffer from limited intrinsic conductivity, requiring hybridization to reach high sensitivity. Within this class, a clear trade-off emerges: NiWO_4_ favors robustness and reproducibility, WSb_2_O_6_-based heterostructures maximize electrocatalytic sensitivity in complex matrices but risk fouling, and CaMoO_4_ systems enable highly selective, low-level detection yet depend heavily on conductive supports. Carbon-based nanomaterials (graphene, CNTs, g-C_3_N_4_) excel in electrical conductivity, surface area, and functionalization flexibility, making them ideal platforms for signal amplification and biomolecule immobilization. However, their limited intrinsic catalytic activity and aggregation/restacking issues can restrict performance unless combined with active materials. In contrast, noble metal nanoparticles provide exceptional catalytic activity and sensitivity but are constrained by high cost, aggregation, and long-term instability, limiting scalability. Emerging 2D materials such as MXenes and transition metal dichalcogenides bridge some of these gaps by offering high conductivity and tunable surface chemistry, yet they introduce new challenges, including oxidation instability (MXenes) and poor baseline conductivity (TMDCs). Similarly, conducting polymers contribute flexibility and biocompatibility but lack long-term environmental stability. Overall, the central limitation across all material systems is the inability to simultaneously optimize conductivity, catalytic activity, stability, and anti-fouling properties. This has driven a clear shift toward hybrid and composite architectures, where synergistic combinations are not optional but necessary to achieve practical sensor performance in real food matrices.

## 5. Target Analytes, Detection Strategies and Real-World Applications

### 5.1. Food Antioxidants

Food antioxidants like tert-butylhydroquinone, a synthetic phenolic compound, are widely used to prevent oxidative rancidity in edible oils, fats, and processed foods, but excessive consumption has been associated with potential health risks, including DNA damage, tumor promotion, and metabolic disturbances, necessitating monitoring to ensure compliance with regulatory limits typically ranging from 100 to 200 mg/kg in oils and fats. Detection challenges include complex lipid-rich food matrices containing numerous interfering compounds such as other phenolic antioxidants, natural polyphenols, tocopherols, and lipid oxidation products; electrochemical behavior similar to that of other phenolic compounds that undergo oxidation at similar potentials; low concentrations requiring high sensitivity, particularly when monitoring at or below regulatory limits; and the need for minimal sample preparation to enable rapid screening as extensive cleanup would negate the speed advantages of electrochemical sensing. SnS nanoflower-modified electrodes achieve detection limits of 0.008 µM through hierarchical morphologies providing high surface area and electrocatalytic enhancement, with successful application to edible oils, including sunflower, olive, and peanut oils, demonstrating recovery rates from 96.5 to 103.2%. F,P-codoped g-C_3_N_4_ sensors achieve even lower sub-nanomolar limits through dual doping, creating synergistic electronic and structural modifications that increase conductivity, create additional active sites, modify band structure, and enhance surface area.

### 5.2. Food Preservatives

**Figure 8:**
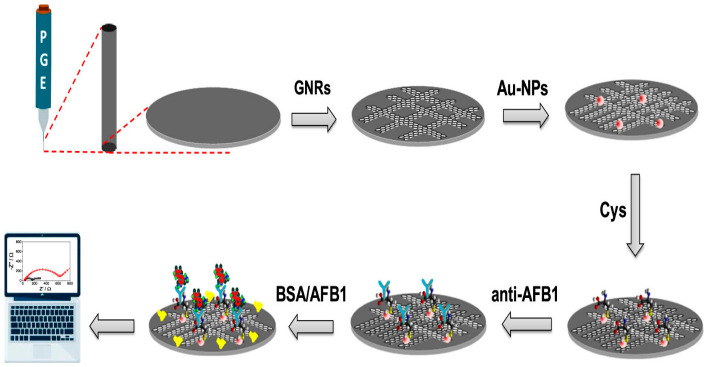

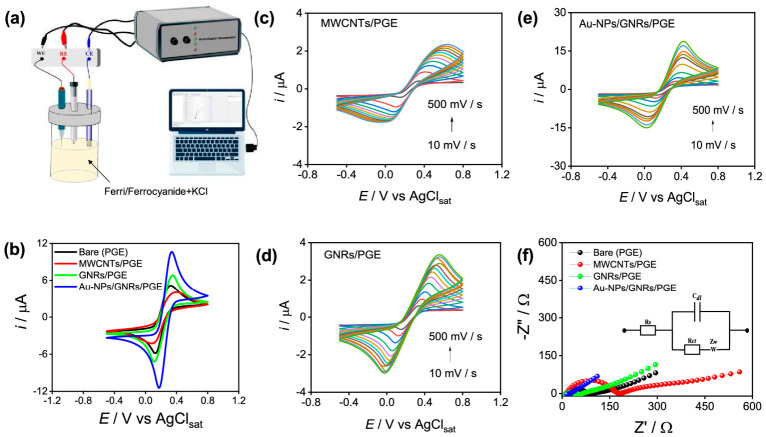
Fabrication and electrochemical characterization of the BSA/anti-AFB1/Cys/Au-NPs/GNRs/PGE biosensor. (**a**) Measurement setup; (**b**–**e**) CVs of modified electrodes; (**f**) EIS spectra of different electrodes. Reproduced with permission from [50].

**Figure 9:**
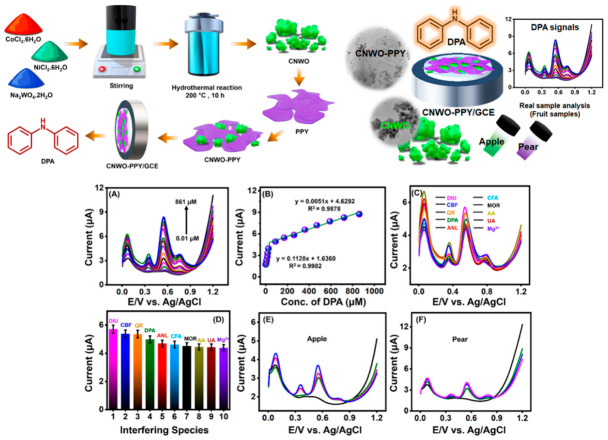
Synthesis of CNWO-PPY nanocomposite and electrochemical behavior of CNWO-PPY/GCE: (**A**,**B**) CVs at different pH; (**C**,**D**) CVs at varying scan rates with DPA; (**E**,**F**) CVs with different DPA concentrations. Reproduced with permission from [61].

Food preservatives and anti-scald agents like diphenylamine are used post-harvest to prevent storage scald in apples and pears, but concerns about potential carcinogenic metabolites and endocrine disruption have led to regulatory scrutiny, with the European Union banning DPA use and other regions maintaining strict maximum residue limits around 10 mg/kg. Au-NPs decorated graphene nanoribbons achieve picomolar detection limits through synergistic properties combining the conductivity and surface area of graphene with the catalytic activity of gold nanoparticles and enhanced edge effects in ribbon geometry (Figure 8) [50]. Real sample analysis of apple and pear extracts with minimal preparation shows recovery rates from 98.2 to 102.5% and good correlation with HPLC reference methods, with correlation coefficients exceeding 0.98, validating reliability for regulatory compliance testing [66,67,68,69,70,71,72,73,74]. Hierarchical cobalt–nickel–tungstate-anchored polypyrrole represents an alternative approach employing ternary metal oxide combined with conducting polymer, achieving similar performance by combining the catalytic activity of mixed metal oxides with the conductivity of the polymer matrix (Figure 9) [75,76,77,78,79].

Although SnS nanoflower-, F,P-codoped g-C_3_N_4_-, AuNP-decorated graphene nanoribbon-, and cobalt–nickel–tungstate/polypyrrole-based electrochemical sensors show excellent sensitivity for detecting food additives such as tert-butylhydroquinone (TBHQ) and diphenylamine (DPA), their practical value depends strongly on the reproducibility of nanomaterial synthesis, storage stability, and repeated measurement stability. Reproducible fabrication is challenging because small variations in precursor ratio, temperature, reaction time, dopant incorporation, nanoparticle size, or composite composition can significantly alter morphology, conductivity, and catalytic activity, leading to batch-to-batch signal variation. Storage stability is another concern, as sulfides may oxidize, polymers may overoxidize or crack, graphene surfaces may adsorb contaminants, and nanoparticles may aggregate or detach during prolonged storage, reducing sensor response over time. Repeated-use stability is especially important for real food samples such as edible oils and fruit extracts, where lipids, sugars, and polyphenols can foul the electrode surface and cause current loss or baseline drift after multiple scans. Therefore, while the reported low detection limits and good recoveries are promising, routine regulatory application requires validation through a low relative standard deviation between batches, retention of ≥90% signal after weeks of storage, and consistent performance over dozens of repeated measurements.

### 5.3. Mycotoxins

Mycotoxins such as aflatoxins pose serious food safety threats, as they are potent carcinogens produced by *Aspergillus* species that can occur in staple foods, including maize, peanuts, tree nuts, and dried fruits. Aflatoxin B1 is classified as a Group 1 human carcinogen by the International Agency for Research on Cancer, with extremely stringent regulatory limits ranging from 2 to 20 µg/kg depending on jurisdiction and food type, reflecting serious health risks, including liver cancer. Electrochemical immunosensor approaches employing antibodies for molecular recognition with electrochemical signal transduction represent the most successful strategy, as the extremely low detection limits and high specificity requirements generally preclude direct electrochemical detection. MXene-based immunosensors achieve detection limits below 0.1 ng/mL through a high surface area providing abundant antibody immobilization sites and excellent conductivity facilitating electron transfer, with signal amplification through enzyme labels, nanomaterial labels, or DNA amplification strategies enabling sub-parts-per-billion detection.

### 5.4. Food Allergens

Food allergens like gliadin proteins trigger celiac disease in approximately 1 percent of the global population and non-celiac gluten sensitivity in broader populations, requiring strict gluten-free diets maintaining gluten levels below 20 ppm. MXene-based gliadin immunosensors achieve detection limits of 0.1 ng/mL, equivalent to approximately 2 ppm gluten, with linear ranges covering regulatory concentrations, response times under 10 min representing 10- to 20-fold time savings compared to ELISA, high selectivity against other proteins, and excellent reproducibility. Real sample validation across wheat flour, baked goods, beer, and fermented products demonstrates recovery rates from 97.5 to 103.5% and good correlation with ELISA reference methods, with R^2^ values exceeding 0.95. Advantages over conventional methods include 10-fold faster analysis, enabling same-shift results for production quality control, significantly lower cost per test due to disposable electrodes, minimal equipment and training requirements, and portability through handheld potentiostat compatibility enabling field testing.

Many of these nanomaterials (such as MXene, graphene and complex metal oxides) require costly precursors, multi-step synthesis routes, controlled atmospheres, hazardous etchants, high-temperature treatments, or lengthy purification steps that can significantly increase production costs at industrial scale. Likewise, electrode fabrication methods commonly used in laboratories—such as drop-casting, spin-coating, hydrothermal growth, or manual slurry deposition—often lack the uniformity, throughput, and automation needed for large-scale manufacturing. Batch-to-batch variability, material waste, substrate compatibility, and integration with roll-to-roll printing or screen-printing processes remain major barriers to commercialization. In addition, long-term storage, packaging, regulatory approval, and quality-control requirements for disposable food sensors are rarely discussed. Consequently, while these nanomaterial-based prototypes demonstrate impressive analytical sensitivity in research settings, the pathway from proof-of-concept devices to affordable, mass-produced commercial sensors remains insufficiently addressed and requires greater focus on scalable synthesis, low-cost fabrication, and manufacturing readiness.

## 6. Smart Packaging, Wireless Sensing, and Future Perspectives

**Figure 10:**
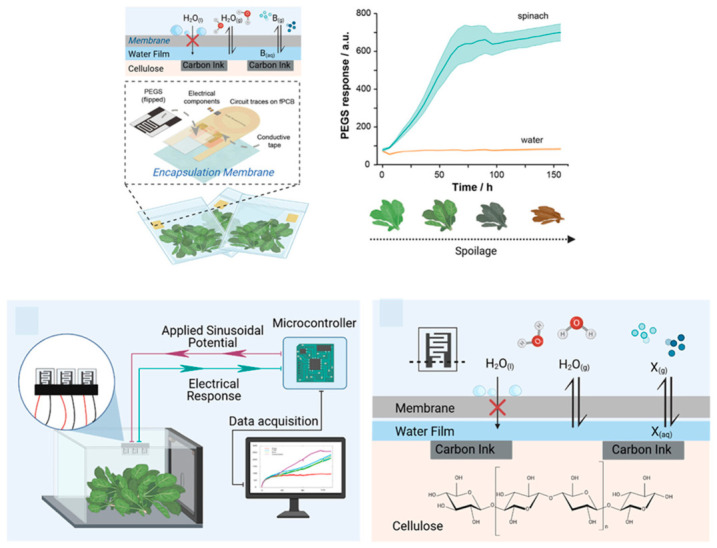
Schematic of a PEGS-based system for monitoring spinach spoilage via conductance changes; illustration of a gas-selective membrane and water vapor condensation enabling gas dissolution. Reproduced with permission from [80].

**Figure 11:**
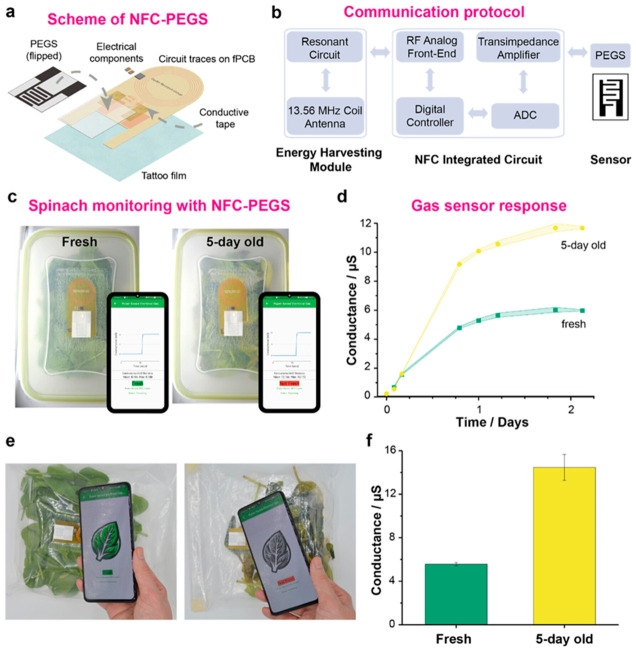
(**a**) Exploded view of an NFC-powered batteryless device; (**b**) block diagram of NFC electronics; (**c**) device with PEGS in food boxes and app interface; (**d**) PEGS conductance over time for fresh vs. spoiled spinach; (**e**) device in food bags with app (user version); (**f**) conductance comparison after 24 h. Reproduced with permission from [80].

Intelligent food packaging represents a fundamental shift from static barrier materials to dynamic, information-rich systems that monitor food quality, communicate status, and enable real-time decision-making throughout supply chains. Near-field communication has emerged as the leading wireless platform due to its operation at 13.56 MHz in the globally unlicensed ISM band, a communication range of 1 to 10 cm, which is sufficient for intentional interaction while preventing unintended reads, passive operation powered by the reader’s electromagnetic field, eliminating the need for batteries, and compatibility with ubiquitous smartphone NFC readers. Food spoilage is accompanied by the generation of volatile organic compounds with specific profiles characteristic of different processes, including trimethylamine indicating seafood spoilage, ammonia indicating protein degradation, hydrogen sulfide indicating anaerobic bacterial growth, and ethanol indicating fermentation [80]. NFC-enabled wireless gas sensor systems employ chemiresistive materials whose resistance changes upon gas exposure, achieving sensitivity at ppm to ppb levels and response times from 1 to 10 min, and reducing the target costs per sensor for commercial viability (Figure 10 and Figure 11).

The true value of smart packaging is realized through effective data management and decision support, with data flow architecture involving sensor measurement, local processing, wireless transmission, cloud storage, predictive modeling using machine learning, alert generation, and supply chain integration. Artificial intelligence (AI) integration substantially expands the capabilities of electrochemical sensing platforms by transforming raw signals into actionable insights. Modern sensors often generate multivariate data (current, potential, impedance, time-resolved responses), which are difficult to interpret using conventional methods. AI-driven pattern recognition techniques—such as machine learning and deep learning—can extract subtle correlations within these complex datasets, enabling more accurate identification of target analytes even in highly heterogeneous food matrices. Beyond detection, AI enables classification of food types and quality grades by learning characteristic electrochemical signatures associated with freshness, contamination levels, or compositional differences. For example, supervised learning models can distinguish between fresh and spoiled products or grade food quality based on deviations in sensor response patterns. In parallel, anomaly detection algorithms can identify unexpected signal variations that may indicate toxic substances, adulteration, or sensor malfunction, providing an additional layer of reliability and safety assurance. AI also contributes to decision optimization, where predictive models integrate sensor outputs with contextual data (e.g., temperature history, storage conditions, supply chain timelines) to generate recommendations. These may include real-time alerts, shelf-life prediction, or optimized inventory management strategies that reduce waste and improve food safety compliance. Overall, AI integration shifts electrochemical sensing from a purely analytical tool to an intelligent, adaptive system capable of continuous learning and autonomous decision support in modern food monitoring applications. Current market status shows adoption primarily in high-value supply chains with strong consumer interest but price sensitivity. Technical challenges include reducing manufacturing costs to commercially viable levels, ensuring reliable and consistent sensor performance, establishing standardized industry-wide production and validation protocols, and enabling seamless integration into existing manufacturing, packaging, and distribution infrastructure. Economic considerations include value proposition justifying costs through waste reduction, supply chain savings through optimized inventory, and consumer willingness to pay premiums [80,81,82,83,84,85,86].

Future directions include next-generation nanomaterials such as two-dimensional materials beyond graphene and MXene, nanostructured perovskites, biomimetic nanomaterials, and quantum dots offering enhanced properties. Artificial intelligence and machine learning integration enable pattern recognition, predictive modeling, sensor optimization, and data fusion. Advanced sensor architectures include wearable and flexible sensors, implantable sensors for continuous in-package monitoring, lab-on-a-chip platforms, and 3D-printed sensors. Wireless and IoT integration enables real-time monitoring across entire supply chains, automated alerts, blockchain integration for traceability, and big data analytics. Sustainability considerations include use of earth-abundant non-toxic materials, biodegradable components, minimal waste generation, energy-efficient operation, and recyclability. Personalized nutrition and health monitoring represent the convergence of food sensors with personal health, enabling real-time dietary tracking, allergen detection, nutrient analysis, and integration with health apps.

## 7. Conclusions

The field of electrochemical food sensors has undergone remarkable transformation driven by the convergence of nanomaterials science, electroanalytical chemistry, wireless technologies, and data analytics. Nanomaterial-based electrochemical sensors have achieved performance levels rivaling or exceeding traditional laboratory methods for many food safety applications, with materials innovation providing unprecedented control over electrode surface properties, enabling detection at ultra-trace levels with excellent selectivity. Smart packaging integration has created pathways for continuous real-time monitoring throughout supply chains, offering transformative potential for reducing waste, preventing illness, and providing transparency. Despite impressive advances, challenges must be addressed, including improving stability, enhancing reproducibility, reducing costs, establishing standards, and demonstrating value.

Key limitations that remain insufficiently addressed include the long-term biocompatibility and stability of antibodies immobilized on MXene surfaces, the susceptibility of enzyme-based sensing layers to degradation in real food environments, and the absence of standardized validation protocols for electrochemical sensors in complex food matrices. Although MXene provides high conductivity and abundant surface functional groups for antibody attachment, prolonged exposure may lead to surface oxidation, changes in binding orientation, loss of bioactivity, or potential cytotoxicity concerns if particles leach into food-contact systems. Similarly, enzyme-based sensors often suffer from denaturation, proteolytic breakdown, pH sensitivity, and inhibition by salts, fats, preservatives, or natural phenolics commonly present in foods, resulting in shortened operational lifetime and signal drift. In addition, performance claims are difficult to compare across studies because there is no universally adopted testing framework for food electrochemical sensors covering sample preparation, matrix-matched calibration, fouling resistance, storage stability, recovery criteria, inter-laboratory reproducibility, or benchmark comparison with reference methods such as HPLC or LC-MS. Addressing these issues is essential for translating promising laboratory sensors into reliable commercial food-monitoring technologies. Looking forward, we envision a food system where continuous monitoring provides real-time quality information, predictive safety enables proactive interventions, and supply chain transparency provides complete traceability, with potential to reduce food waste by 30 to 50 percent and fundamentally transform food safety management. The synergy between materials science, nanotechnology, and electroanalytical chemistry has created a powerful platform for addressing critical food safety challenges, with continued collaboration of researchers, industry, regulators, and end users essential to realizing this vision and delivering benefits to society.

## Figures and Tables

**Table 1:** Carbon-based vs. non-carbon nanomaterials in electrochemical food sensors.

Nanomaterial	Key Advantages	Key Disadvantages	References
Graphene/Graphene oxide (GO, rGO)	Exceptional electrical conductivity; very high surface area; easy functionalization; strong π–π interactions with analytes	Restacking reduces active surface area; batch variability; possible aggregation	[31,33]
Carbon nanotubes (CNTs)	Excellent electron transfer kinetics; high mechanical strength; high aspect ratio enhances sensitivity	Difficult dispersion; metallic vs. semiconducting variability; potential toxicity concerns	[32,35,38,39]
Carbon nitride (g-C_3_N_4_)	Metal-free catalyst; good chemical stability; tunable electronic structure; low cost	Lower conductivity than graphene; limited active sites without modification	[30]
Carbon quantum dots (CQDs)	Easy synthesis; good biocompatibility; fluorescence + electrochemical dual functionality	Lower conductivity; limited catalytic activity compared to metals	[30]
Metal nanoparticles (Au, Ag, Pt)	Excellent electrocatalytic activity; high sensitivity; easy surface functionalization (e.g., thiols on Au)	High cost (especially Au, Pt); susceptibility to aggregation; stability issues	[27,37]
Metal oxide nanostructures (ZnO, TiO_2_, NiO, SnO_2_)	High chemical stability; good catalytic activity; tunable band gap; low cost	Poor intrinsic conductivity; often require composites with carbon	[31,34]
MXenes (e.g., Ti_3_C_2_T_x_)	Metallic conductivity; hydrophilic surfaces; excellent electrochemical performance; high surface area	Oxidation instability; synthesis complexity; limited long-term stability	[26]
Transition metal dichalcogenides (MoS_2_, WS_2_)	Layered structure; tunable bandgap; catalytic edge sites	Low conductivity in bulk form; restacking issues	[23,24,25]
Conducting polymers (PANI, PPy, PEDOT)	Good electrical conductivity; flexibility; easy synthesis; good biocompatibility	Poor long-term stability; environmental sensitivity (pH, temperature)	[33]

**Table 2:** Comprehensive critical summary of nanomaterials for electrochemical food sensing.

Material	Representative Systems	Typical Performance	Key Strengths	Critical Limitations	Best-Suited Applications	When Not to Use	Ref.
Binary metal oxides	SnS	µM–nM LOD; wide linear range	High surface area; good catalytic activity; stable	Moderate conductivity; limited selectivity	Antioxidants (e.g., TBHQ) in oils	Ultra-trace detection in complex biological matrices	[25]
Ternary metal oxides	NiWO_4_	nM–µM LOD; stable response	Excellent stability; mixed redox activity; reproducible	Lower sensitivity vs. composites	Routine, long-term sensing	Applications requiring ultra-low LOD	[23]
	WSb_2_O_6_/f-CNF	nM LOD (~9–11 nM)	Strong electrocatalysis; heterojunction-enhanced sensitivity	Fouling risk; matrix interference; complex synthesis	Complex matrices (milk, dairy)	Long-term reusable sensors without regeneration	[24]
	CaMoO_4_/P-CNT	sub-nM–nM LOD; high sensitivity (>1.9 µA µM^−1^ cm^−2^)	Strong analyte interaction; high sensitivity	Poor intrinsic conductivity; hybrid-dependent	Target-specific sensing (vanillin, phenolics)	Simple, low-cost sensor designs	[40]
Carbon-based materials	Graphene, CNTs	nM–pM (with modification)	Excellent conductivity; high surface area; versatile	Low intrinsic catalytic activity; aggregation	Biosensors; signal amplification platforms	Standalone catalytic sensing	[41,49]
g-C_3_N_4_	Doped g-C_3_N_4_	sub-nM LOD	Metal-free; tunable; stable	Low conductivity without doping	TBHQ, organic pollutants	Fast-response sensing without modification	[45,46]
Noble metals	Au, Pt NPs	pM–nM LOD	Exceptional catalytic activity; high sensitivity	Expensive; aggregation; poor long-term stability	Ultra-trace detection; enzyme-free sensing	Scalable, low-cost applications	[50]
MXenes	Ti_3_C_2_T_x_	ng/mL–nM LOD	Metallic conductivity; fast response; large surface	Oxidation instability; synthesis sensitivity	Protein detection (gliadin), rapid assays	Long-term storage or harsh environments	[26,56]
TMDCs	MoS_2_, WS_2_	nM LOD	Active edge sites; tunable bandgap	Poor conductivity; restacking	Catalysis-assisted sensing	High-speed or high-current applications	[66]
Conducting polymers	Polypyrrole composites	nM LOD	Flexible; biocompatible; easy synthesis	Environmental instability; degradation	Wearable sensors; biosensors	Harsh chemical environments	[61]
Hybrid/composites	Metal oxide–carbon, metal–carbon	pM–nM LOD	Synergistic conductivity + catalysis + stability	Complex fabrication; reproducibility issues	Real-world food sensing (best overall)	Situations requiring ultra-simple fabrication	[37,38,39]

## Data Availability

No new data were created or analyzed in this study. Data sharing is not applicable to this article.
